# Population Genetic Structure Within and among Seasonal Site Types in the Little Brown Bat (*Myotis lucifugus*) and the Northern Long-Eared Bat (*M*. *septentrionalis*)

**DOI:** 10.1371/journal.pone.0126309

**Published:** 2015-05-05

**Authors:** Laura N. L. Johnson, Brenna A. McLeod, Lynne E. Burns, Krista Arseneault, Timothy R. Frasier, Hugh G. Broders

**Affiliations:** 1 Biology Department, Saint Mary’s University, Halifax, Nova Scotia, Canada; 2 Nova Scotia Museum of Natural History, Halifax, Nova Scotia, Canada; 3 Biology Department, Dalhousie University, Halifax, Nova Scotia, Canada; Southern Illinois University, UNITED STATES

## Abstract

During late summer and early autumn, temperate bats migrate from their summering sites to swarming sites, where mating likely occurs. However, the extent to which individuals of a single summering site migrate to the same swarming site, and vice versa, is not known. We examined the migratory connectivity between summering and swarming sites in two temperate, North American, bat species, the little brown bat (*Myotis lucifugus*) and the northern long-eared bat (*Myotis septentrionalis*). Using mitochondrial and microsatellite DNA markers, we examined population structuring within and among summering and swarming sites. Both species exhibited moderate degrees of mitochondrial DNA differentiation (little brown bat: *F_ST(SWARMING)_*= 0.093, *F_ST(SWARMING)_*= 0.052; northern long-eared bat: *F_ST(SWARMING)_*= 0.117, *F_ST(SWARMING)_*= 0.043) and little microsatellite DNA differentiation among summering and among swarming sites. Haplotype diversity was significantly higher at swarming sites than summering sites, supporting the idea that swarming sites are comprised of individuals from various summering sites. Further, pairwise analyses suggest that swarming sites are not necessarily comprised of only individuals from the most proximal summering colonies.

## Introduction

Migration allows species inhabiting seasonal environments (which vary in temporal and spatial resource variability) to exploit ephemeral resources [1,2]. Although migration is a taxonomically widespread phenomenon, the extent of connectivity between seasonal sites remains unknown for most species. Many species differ in the extent to which individuals of a single summering site migrate to the same wintering site, and vice versa, and such patterns may vary with age and sex [3]. In some species, most individuals occupy a single site during the breeding season and migrate to separate foraging sites (e.g., gray whale, *Eschrichtius robustus*) [4], whereas in other species several sites may be used during the breeding season with all individuals converging at a single foraging site (e.g., several species of sea turtles) [5]. Understanding migration dynamics and the extent to which populations have a spatial affinity amongst seasonal sites is important for management, as it can help identify which regions are critical habitat for local populations.

With cryptic species that are capable of long distance movement, direct observation of migration patterns is often costly or logistically difficult. For such species genetic analyses can provide a practical approach to infer migration dynamics. Specifically, comparing the genetic characteristics of individuals from various seasonal sites can provide information on population structuring and the degree of connectivity both within and across seasonal site types. Moreover, different molecular markers can be used to assess different aspects of migration. While mitochondrial and Y-chromosome markers can be used to compare migration patterns between sexes, nuclear markers can show how these patterns influence population-wide patterns of structuring e.g. [6–10].

Many studies on temperate bats have focused on how behaviour and habitat use change with the seasons. However, for many, the migratory connectivity remains largely unknown, as their nocturnal behaviour and vagility typically precludes direct detection of migration. For bats that are year-round residents of temperate areas, individuals migrate between summering areas and winter hibernacula [11–13]. Upon emergence in spring, individuals migrate to summering areas where they roost in trees or manmade structures [14,15]. Females form maternity colonies, where they give birth and rear offspring while males typically remain solitary [16,17]. In late summer to mid-autumn, both sexes migrate to hibernacula where large numbers of bats congregate prior to hibernation [18–20]. Both behavioural and genetic data support the hypothesis that, for some species, mating occurs during this ‘swarming’ season [21–25]. Swarming is also thought to potentially facilitate information transfer about important sites from mothers to offspring and conditions of hibernacula [19,26].

Genetic studies of temperate bats suggest that females exhibit philopatry to maternity colonies, but the degree of philopatry varies considerably among species [9,27–29]. For example, Bechstein’s bats (*Myotis bechsteinii*) exhibit strict philopatry, where females return to their natal maternity site. In contrast, the big brown bat (*Eptesicus fuscus*) exhibits high mitochondrial diversity within maternity colonies coupled with fewer observations of returning mothers and daughters, suggesting lower philopatry to maternity roosts [7]. Male philopatry to summering sites has only been recorded in two bat species; the brown long-eared bat (*Plecotus auritus*) [30] and the Natterer’s bat (*M*. *nattereri*) [23], where both sexes appear to exhibit philopatry.

Several studies suggest that swarming sites are comprised of bats from multiple summering areas [22–25,31]. In the Natterer’s bat, for example, swarming sites have a catchment area that includes individuals from surrounding summering sites [32]. Additionally, individuals may visit multiple swarming sites in a single night, suggesting that catchment areas for different swarming sites may overlap [32,33]. However, for most temperate bat species the migratory characteristics and population structuring across swarming and summering sites remains unknown.

The little brown bat (*M*. *lucifugus*) and the northern long-eared bat (*M*. *septentrionalis*) are widely distributed across North America and exhibit seasonal movement patterns typical of bats that are year-round residents of temperate areas [14,34]. Female little brown bats reside in colonies that typically roost in manmade structures, tree cavities, or other natural crevices during the summer (consisting of 10s -100s of individuals) [14]). Female northern long-eared bats may also roost in manmade structures but are more forest dependent, typically using tree cavities and exfoliating bark as summer roosts [15,34,35]. Additionally, because both species exhibit roost switching throughout summer, inhabiting various roosts in a single summer [15,36], it is unclear how many individuals represent a single maternity colony. Non-reproductive females and males of both species roost individually or within small groups [14,35].

While both species are regional migrants, the northern long-eared bat is known to travel up to 100 km between summering and swarming/hibernation sites [34], while the little brown bat can migrate up to at least 650 km between seasonal sites [19,37,38]. Both species exhibit similar patterns of low overall genetic structuring (e.g. [39,40]) and some degree of female philopatry [9,29,41]. However, given their different migratory patterns and forest dependence, it could be expected that the northern-long eared bat will exhibit a greater degree of population structuring than the little brown bat.

Here we use mitochondrial (mtDNA) and nuclear (nDNA) DNA to examine genetic structuring of the little brown bat and northern long-eared bat at summering and swarming sites to infer movement patterns between seasonal sites. Specifically, we predicted that both male and female bats would exhibit philopatry to summering regions and that swarming sites would be used by bats from several summer roosts. Therefore, we expected greater genetic differentiation among summering regions, and higher degrees of relatedness between individuals within summering sites than would be expected if there was no summer site affinity. Further, since it was expected that bats from several summering sites would use the same swarming sites, we predicted there would be greater genetic diversity at swarming sites than summering sites.

## Materials and Methods

### Sample Collection, Extraction and Preparation

**Fig 1:**
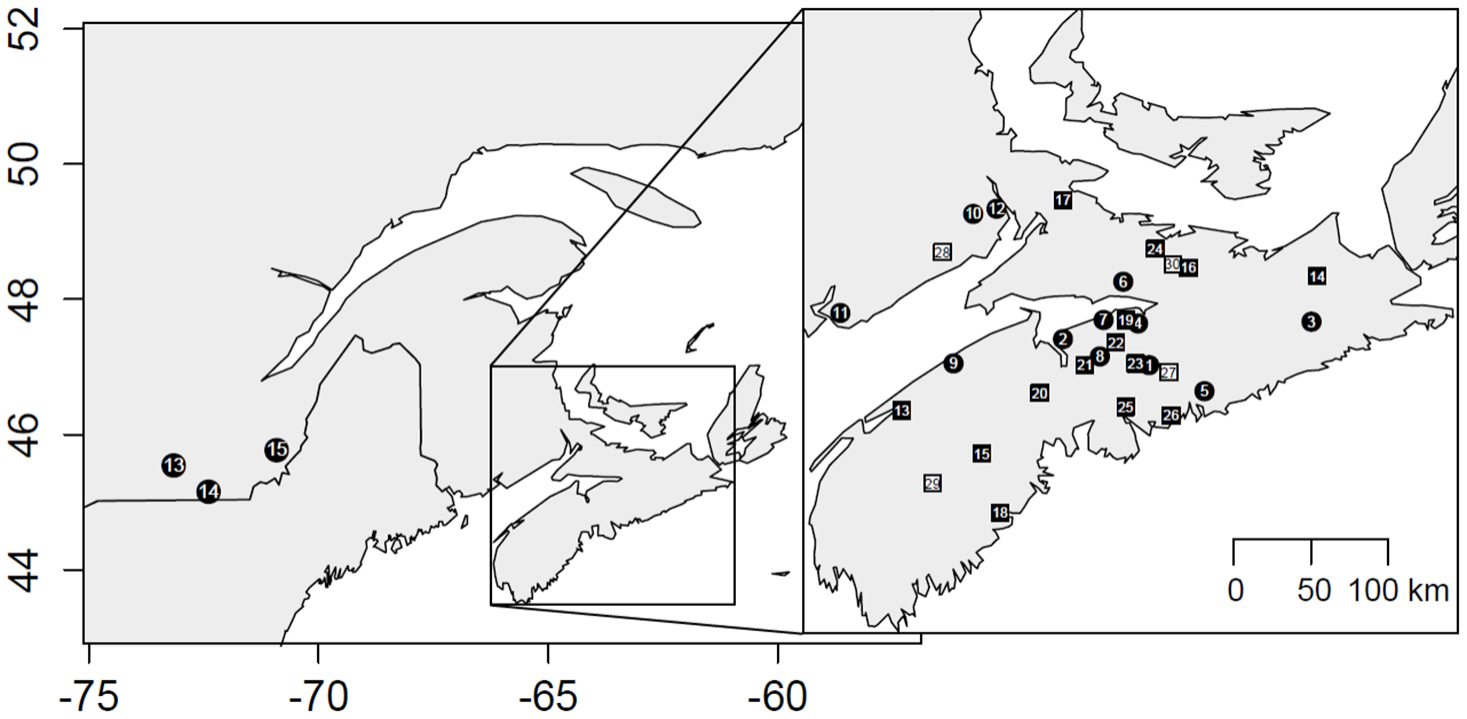
Map of the little brown bat (*Myotis lucifugus*) and northern long-eared bat (*M*. *septentrionalis*) swarming (circles) and summering (squares) sites. Little brown bat summering sites are represented by black squares and white squares represent northern long-eared summering sites. Both species were sampled from all swarming sites excluding Vault Cave (Site 9). Tissue samples were collected between 1999–2012 across Quebec, New Brunswick and Nova Scotia, Canada.

We collected tissue samples of adult little brown bats between 2008–2012 from fifteen swarming sites and fourteen maternity roosts in New Brunswick, Nova Scotia, and Quebec, Canada (n = 1322; 542 males, 780 females; Fig 1; S1 Table). For northern long-eared bats, we collected samples between 1999–2012 from eleven swarming sites and four summering areas in New Brunswick and Nova Scotia, Canada (n = 457; 242 males, 215 females; Fig 1). Swarming site sample collection was conducted from mid-August to October while summering samples were collected from May to early August. We used harp traps (Ausbat Research Equipment, Lower Plenty, Victoria, Australia) or mist nets (Avinet, Dryden, New York, USA), set at the entrance of underground sites to capture bats during swarming. Similarly, during the summer season we used a combination of harp traps and mist nets to capture little brown bats as they emerged from roosts in buildings, and to capture northern long-eared bats along forest trails. We identified individuals to species and determined their sex and age (either adult or young-of-the-year [42]). We collected 3–5 mm diameter tissue samples from the plagiopatagium or uropatagium from each wing using forceps and cuticle scissors or a 3 mm diameter biopsy punch [43,44]. We released all bats at the site of initial capture. We sterilized sampling equipment with 95% ethanol between samples, and preserved tissue samples as per Burns et al. [40]. Our study was carried out following the annual approval from the Saint Mary’s University Animal Care Committee (protocol numbers: 08–20, 09–24, 10–11, 11–18, 12–17 and 13–15) and under permits from the Nova Scotia Department of Natural Resources and the New Brunswick Department of Natural Resources. Samples from Quebec were provided by the Quebec Ministère des Ressources naturelles et de la Faune (J. Mainguy and A. Meschede). We extracted genomic DNA from both wing samples following a standard phenol:chloroform technique [45] followed by ethanol precipitation as per Burns et al. [40].

### Mitochondrial DNA Analysis

We amplified approximately 400 bp of hypervariable region II (HVII) of the mtDNA control region as per Burns et al. [40] with the exception of an altered annealing temperature for the northern long-eared bat (48°C). To degrade remaining dNTPs and excess primers in the amplified solution, we performed 5.78 μl reactions containing: 5 μl amplified DNA, 1.29X Antarctic phosphatase buffer (50 mM Bis-Tris-Propane-HCl, 1 mM MgCl_2_, 0.1 mM ZnCl_2_, pH 6.0), 0.1 U/μl Antarctic phosphatase (New England Biolabs), and 0.123 U/μl exonuclease I for each sample. We then incubated samples for 15 minutes at 37°C and for 15 minutes at 80°C. To conduct Sanger sequencing [46], we sent our PCR products to Macrogen Inc. (Seoul, Korea). We initially sequenced a subset of samples in both directions to determine which primer provided a more reliable sequence (22 for the little brown bat and 40 for the northern long-eared bat). After determining that there were no sequence discrepancies, the remaining samples were sequenced in one direction (the little brown bat with KAHVII, the northern long-eared bat with L16517).

We manually trimmed and edited each sequence using MEGA v5.1 [47], aligned sequences using CLUSTAL W [48] implemented in MEGA, and then reviewed alignments by eye. We imported sequences into FaBox 1.41 to identify individuals with identical sequences and assign haplotypes to each sequence [49]. Modelgenerator v85 was used to determine the most appropriate model of molecular evolution [50]. We then used that model (Tamura and Nei for both species) [51] to estimate the transition/transversion ratio and the α parameter associated with the gamma distribution for rate heterogeneity of substitutions across sites using TREE-PUZZLE 5.2 [52].

To assess genetic differentiation among sites, we conducted classical analyses of population structure, where individuals were assigned *a priori* to sample sites. First, we estimated haplotype diversity (*h*) and nucleotide diversity (π) for each sampling site using Arlequin v 3.5 [53]. To determine if swarming and summering sites exhibit significant differences in haplotype diversity, which would be expected if multiple summering colonies congregate at a single swarming site, we performed a two tailed, Mann-Whitney U-test [54]. We conducted analyses of molecular variance (AMOVA) for each site type, independently, to assess the genetic differentiation, and degree of philopatry towards summering sites or fidelity to swarming sites, respectively, using Arlequin. AMOVA results include estimates of genetic variation within and among sample sites, as well as *F*_*ST*_ estimates across all sites. *F*_*ST*_ values were calculated by relating the amount of genetic variation among sites to variation over all the sites analyzed [55]. We assessed the significance of *F*_*ST*_ values using 1000 permutations and adjusted for multiple analyses using Bonferroni correction [56]. Additionally, we estimated pairwise *F*_*ST*_ values among seasonal sites using Arlequin. This allowed us to assess the degree to which bats from summering sites have an affinity to swarming sites. To determine if movement patterns were influenced by spatial scale, we conducted a spatial analysis of genetic structure by performing an isolation by distance analysis (IBD) for each site type using a Mantel test implemented in the web-based IBDWS v 3.32 [57]. Our genetic distance data was transformed using Rousset’s correction [58]. Isolation by distance was used to determine the relationship between calculated pairwise *F*_*ST*_ and pairwise geographic distances (km) between collection sites.

### Microsatellite DNA Analysis

For samples of both species, we amplified ten nDNA microsatellite loci, developed for *M*. *lucifugus* [59] (S2 Table). We carried out PCR amplification in 20 μL reaction volumes containing 10 ng template DNA, 1X PCR buffer (GoTaq Flexi, Promega Inc.), 0.2 mM of each dNTP (Invitrogen), 1.5 mM MgCl_2_ (Promega Inc.), 0.08–0.2 μM of each primer (S2 Table), 0.16mg/mL of bovine serum albumin (Sigma Aldrich), and 0.05 U/μL of GoTaq Flexi DNA polymerase (Promega Inc.). PCR cycling conditions were the same as those for mtDNA analysis, however, annealing temperatures varied between multiplexes (S2 Table). We prepared samples for capillary electrophoresis and fragment analysis on an Applied Biosystems 3500xL Genetic Analyzer by combining 2 μL of PCR product diluted in deionized water, 0.25 μL GeneScan-600 LIZ size standard (ABI) and 10 μL HIDi formamide (ABI).

We examined electropherograms by eye and binned and scored allele sizes using GeneMarker software 2.0 (SoftGenetics). Further analyses using nDNA were only conducted on individuals containing alleles for five or more loci. To determine the number of alleles, null allele frequency estimates, observed and expected heterozygosities, and extent of deviation from Hardy—Weinberg Equilibrium (HWE) we used Cervus 3.0 [60]. Additionally, we calculated inbreeding coefficients (*F*_*IS*_) using SPAGeDi 1.3 [61] and conducted global tests of heterozygote excess/deficiency [62] using Genepop 4.2.1 [63].

As with the mtDNA data, we used classical assessments to estimate genetic differentiation between sites and site types, and to infer the extent of site fidelity. To examine site fidelity and genetic differentiation between summering sites and between swarming sites, we estimated *F*_*ST*_ of summering and swarming sites independently, using the methods described in Weir & Cockerham [64], as implemented in Genepop 4.2.1 [63], with significance estimated based on 1000 permutations, and adjusted using Bonferroni correction [56]. To complement mtDNA analyses, we obtained pairwise *F*_*ST*_ values to further infer the affinity between bats from summering sites and swarming sites. As well, we conducted an IBD analysis between microsatellite and geographic distance data using a Mantel test implemented in IBDWS on summering and swarming sites separately.

### Analysis of Relatedness

If females are philopatric to summering sites, a greater level of relatedness should occur between individuals at these sites relative to between females at different sites. To determine if relatedness was high within summering sites, we estimated the relatedness of bats within and among sites using STORM v. 2.0 [65]. For comparison, we also analyzed relatedness within and among swarming sites. For these analyses, within-group relatedness was estimated for each group. Then, individuals were randomly shuffled between groups while keeping each group size constant. Within-group relatedness was then estimated for each of 1000 shuffling iterations. If females show fidelity to each site type, then within-group relatedness should be significantly higher in the observed groups than in those representing random samples from the population.

### Overall Population Structuring

To assess overall population structuring within each species (including all samples, from both site types), we used two clustering methods that do not require *a priori* assignment of individuals to groups. Instead, the number of genetically differentiated clusters was estimated from the sample set by probabilistically assigning individuals to groups using two methods. First, we used STRUCTURE 2.3.4 [66], which implements a Bayesian approach to estimate the number of clusters (*K*) and assign individuals to those clusters [67]. Assumptions are that loci are unlinked and populations are in Hardy-Weinberg Equilibrium (HWE). The analyses were conducted permitting admixture and for allele frequencies to be correlated across clusters. Values of *K* considered were based on the total number of sampled swarming sites for each species (the little brown bat: *K* = 1–15 and the northern long-eared bat: *K* = 1–11). We used a burn-in of 50,000 Markov Chain Monte Carlo steps, followed by 2,000,000 steps of recorded data. The program was run for 10 iterations of each *K*. To estimate the probability of each *K*, we examined the log probability for each *K*, and the second order rate of change of this probability using the methods described in Evanno et al. [68] as implemented in STRUCTURE HARVESTER [69].

Structuring was also examined using discriminant analysis of principal components (DAPC) using the R package ‘adegenet’ [70,71]. Adegenet uses a multivariate analysis that does not rely on the assumption of HWE, the absence of linkage disequilibrium, or specific models of molecular evolution to identify clusters within genetic data [72]. Here, genetic data were first collapsed into a smaller number of uncorrelated principle components. We used *a*-score optimization to determine the optimal number of principal components to maximize power of discrimination while also minimizing the risk of over-fitting (29 for the little brown bat and 13 for the northern long-eared bat), after which discriminant analysis of principle components was conducted. To examine the variable allelic contributions (or ‘loading’) of each microsatellite locus, we used the ‘loadingplot’ command. Bayesian Information Criterion (BIC) was used to determine the most appropriate value of *K* and individuals were assigned to clusters using DAPC. To examine whether observed clustering could have occurred by chance, we simulated 500 data sets of unrelated individuals for each species based on observed allele frequencies. Each dataset consisted of the same number of individuals as the actual datasets, with the same proportion of missing data at each locus, except simulated individuals were known to be unrelated and from a single gene pool. These random datasets were run through adegenet to examine what patterns may be identified even if no real structuring was present.

## Results

### Mitochondrial DNA Analysis

We obtained mitochondrial DNA control region sequences from 1,234 individuals (819 little brown bats, 415 northern long-eared bats). Sequences were trimmed to 294 bp for little brown bats and 297 bp for northern long-eared bats. We identified 121 little brown bat haplotypes (GenBank accession numbers KP27334-KP273454) and 98 northern long-eared bat haplotypes (GenBank accession numbers KP273236-KP273333). In these species, only 3 (little brown bat) and 2 (northern long-eared bat) haplotypes were identified with a frequency >10% (S2 Table). The number of haplotypes per sampling site varied from 3–19 in little brown bats and 4–20 in northern long-eared bats (S3 Table).

**Fig 2:**
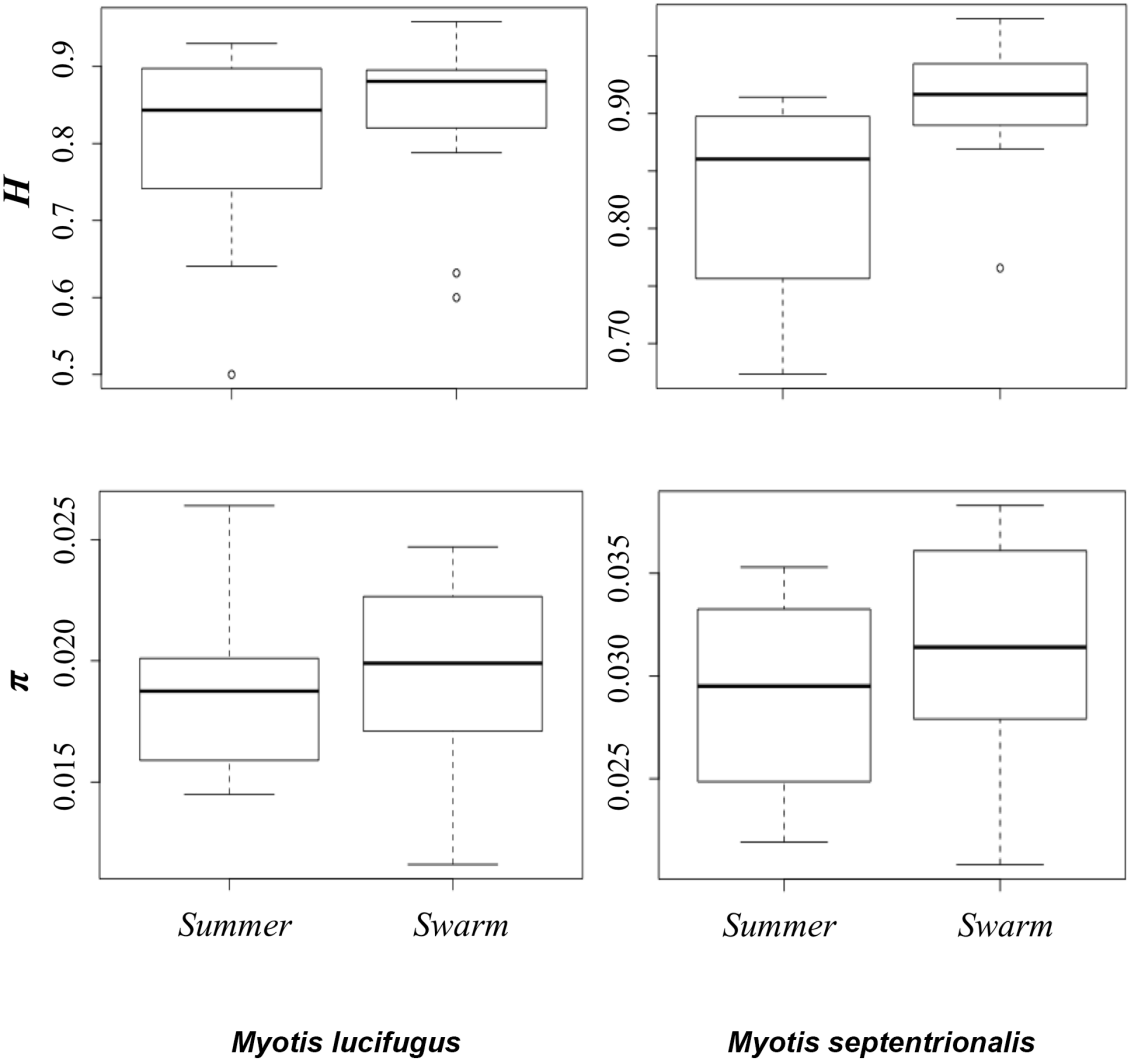
Little brown bat (*Myotis lucifugus*) and northern long-eared bat (*M*. *septentrionalis*) haplotype (*h*) and nucleotide diversity (π). Shown are the results for both summering and swarming sites.

Haplotype diversity (*h*) was not significantly different between summering and swarming sites for either species (little brown bat: *h*_(SWARMING)_ = 0.845, *h*_(SUMMERING)_ = 0.812, Mann-Whitney U-test; *p* = 0.267; northern long-eared bat: *h*_(SWARMING)_ = 0.910, *h*_(SUMMERING)_ = 0.827, Mann-Whitney U-test; *p* = 0.142) (S4 Table; Fig 2). Similarly, no significant difference of nucleotide diversity (π) was identified between site types for both species (little brown bat: π_(SWARMING)_ = 0.020, π_(SUMMERING)_ = 0.019, Mann-Whitney U-test; *p* = 0.484 northern long-eared bat: π_(SWARMING)_ = 0.031, π_(SUMMERING)_ = 0.029, Mann-Whitney U-test; *p* = 0.568; Fig 2).

The AMOVAs conducted for both summering and swarming sites suggested the majority of genetic variation (88.35–95.69%) was attributable to differences among individuals within a site, while a small amount of the variation was explained by differences among sites (4.31–11.65%; Table 1). In the little brown bat the percent of genetic variation explained by differences among summering sites was 1.8x greater than among swarming sites, while in the northern long-eared bat summering site variation was 2.7x greater (Table 1). *F*_*ST*_ values showed greater differentiation among summering sites than swarming sites (little brown bat: *F*_*ST(SUMMER)*_ = 0.093, *p*<0.0001, *F*_*ST(SWARMING)*_ = 0.052, *p*<0.0001; northern long-eared bat: *F*_*ST(SUMMER)*_ = 0.117, *p*<0.0001, *F*_*ST(SWARMING)*_ = 0.043, *p*<0.0001; Table 1). Pairwise *F*_*ST*_ values for little brown bat summering sites resulted in 27 of 91 significant values after we conducted Bonferroni correction (S5 Table). Of these, 10 included site 16 (Earltown, NS). For northern long-eared summering sites, all pairwise *F*_*ST*_ values were significantly different from one another (S5 Table). Pairwise *F*_*ST*_ comparisons between little brown bat swarming sites resulted in 25 significant values out of 105 (S5 Table) and 11 of 55 were significant for northern long-eared bat swarming sites (S5 Table).

**Table 1:** AMOVA results for the partitioning of mtDNA of little brown bat (*M*. *lucifugus*) and northern long-eared bat (*M*. *septentrionalis*) variation across sample site types in eastern Canada and nDNA global F_ST_ across sample site types, as well as all sites.

*Myotis lucifugus*						
	mtDNA				nDNA	
Site Type	% Variation		*F*_*ST*_	p-value	*F*_*ST*_	p-value
	Among	Within				
Summering	9.36	90.64	0.093	<0.0001	0.0030	0.002
Swarming	5.20	94.80	0.052	<0.0001	0.0003	0.018
All Sites	7.34	92.66	0.073	<0.0001	0.0010	<0.001
***Myotis septentrionalis***						
	**mtDNA**				**nDNA**	
**Site Type**	**% Variation**		***F*_*ST*_**	**p-value**	***F*_*ST*_**	**p-value**
	**Among**	**Within**				
Summering	11.65	88.35	0.117	<0.0001	0.007	<0.0001
Swarming	4.31	95.69	0.043	<0.0001	0.001	0.02
All Sites	6.17	93.83	0.062	<0.0001	0.003	<0.0001

Of 255 pairwise comparisons between summering and swarming sites conducted for the little brown bat, 85 were significant (*F*_*ST*_ values varied from 0.056–0.38) after Bonferroni correction (S6 Table). Summering site 16 had the greatest number of significant *F*_*ST*_ values when compared with swarming sites (12 of 15 comparisons), while swarming sites 14 (Mine-aux-Pipistrelles, QC) and 13 (Mont Saint Hilaire, QC) had the greatest number of significant *F*_*ST*_ values when compared with summering sites (11 of 14 comparisons). Of pairwise comparisons between northern long-eared bat summering and swarming sites, 3 of 44 comparisons were significant, all including summering site 29 (Kejimkujik National Park) (S6 Table).

The IBD analysis based on mtDNA data suggested a positive correlation between *F*_*ST*_ and geographic distance for little brown bat swarming sites (*r* = 0.319, *p* = 0.017). However, we did not find a correlation for northern long-eared bat swarming sites (*r* = 0.016, *p* = 0.437). We also found no correlation for summering sites of both species (little brown bat: *r* = 0.197, *p* = 0.163; northern long-eared bat: *r* = 0.519 *p* = 0.173).

### Microsatellite Analysis

We obtained nDNA genotypes from 1,311 little brown bats and 449 northern long-eared bats. For both species, 9 out of 10 loci exhibited significant heterozygote deficiency (*p* < 0.05). This deficiency, across almost all loci, was expected, and is consistent with a Wahlund effect due to the presence of population structure, as opposed to genotyping errors. The microsatellite data are available from the Dryad Digital Repository (doi:10.5061/dryad.47nm0).

*F*_*ST*_ values for each site type were significant but low for both species, with values being 7–10 times greater for summering than swarming sites (little brown bat: *F*_*ST(SUMMER)*_ = 0.003, *p* = 0.002, *F*_*ST(SWARMING)*_ = 0.0003, *p* = 0.018; northern long-eared bat: *F*_*ST(SUMMER)*_ = 0.007, *p*<0.0001, *F*_*ST(SWARMING)*_ = 0.001, *p* = 0.02). Pairwise comparisons conducted between summering and swarming sites for the little brown bat identified one significant value (between summering site 17 Martock, NS and swarming site 12 Whites Cave, NB), however, the genetic differentiation was low (*F*_*ST*_ = 0.0003; S6 Table). Pairwise *F*_*ST*_ comparisons of the northern long-eared bat identified 19 out of 44 significant *F*_*ST*_ values, however all but three had a *F*_*ST*_ value below 0.01 (S7 Table).

Our examination of IBD suggested a positive correlation between *F*_*ST*_ and geographic distance of little brown bat summering sites (*r* = 0.288, *p* = 0.002), and no correlation for little brown bat swarming sites (*r* 0.032, *p* = 0.384). We found no correlation among swarming sites (*r* = 0.120, *p* = 0.173) or summering sites (*r* = -0.401, *p* = 0.796) for the northern long-eared bat.

### Analysis of Relatedness

Observed average relatedness within swarming sites and summering sites were not significantly different from random expectations for both species (little brown bat: *r*_*(SWARMING)*_ = -0.007, *p* > 0.1, *r*_*(SUMMER)*_ = -0.012, *p* > 0.5; northern long-eared bat: *r*_*SWARMING)*_ = -0.017, *p* > 0.1, *r*_*(SUMMER)*_ = -0.026, p > 0.5). Although relatedness was not significant at either site for both species, the average relatedness at swarming sites was 0.65–1.60% lower than at summering sites for both species.

### Overall Population Structuring

**Fig 3:**
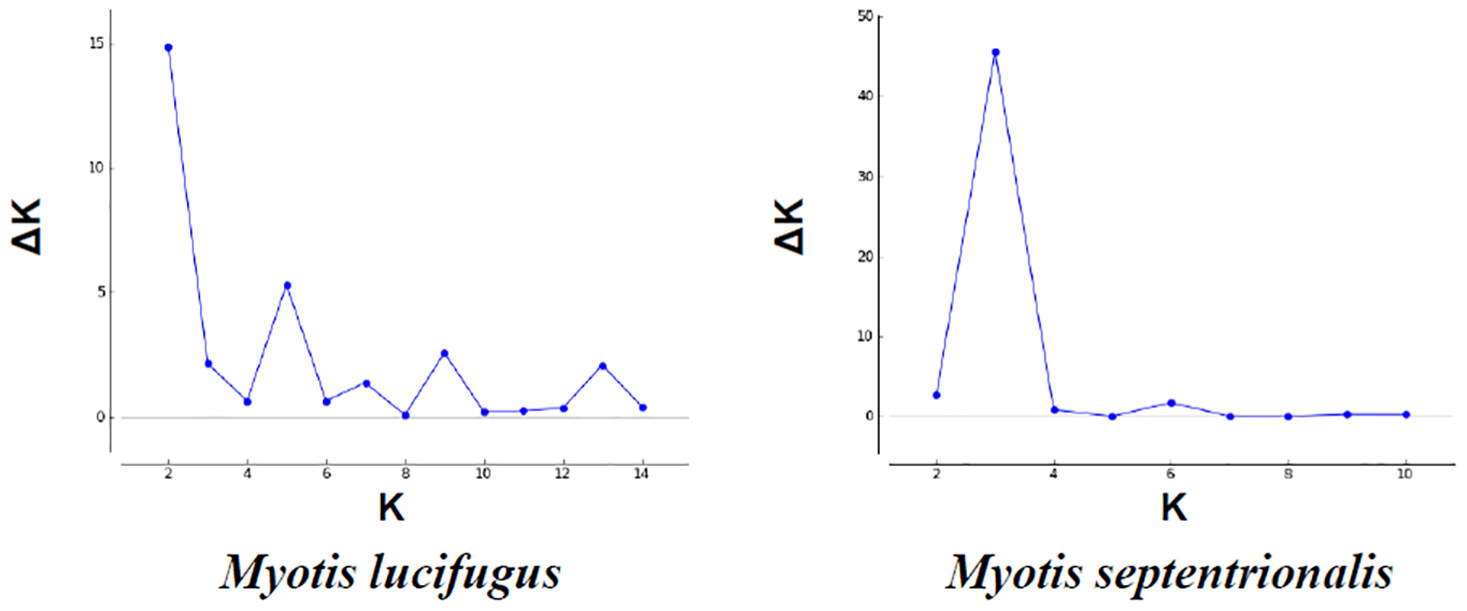
Probability graphs of *K* in little brown bat (*Myotis lucifugus*) and northern long-eared bat (*M*. *septentrionalis*). Plot of mean probability of *ΔK* as calculated by Evanno et al. [69] to detect greatest likelihood of *K*.

Analyses of the second order rate of change for the probabilities of *K*, as determined using STRUCTURE HARVESTER, suggests that the value of K with the highest probability is 2 for the little brown bat (Fig 3). Although calculations for the second order rate of change cannot be performed on *K* = 1, examination of STRUCTURE results and the log probability for each *K*, as determined by STRUCTURE HARVESTER, further suggested that *K* = 2. Assignment probabilities were not symmetrical, with over 60% of individuals being assigned, with a probability ≥70%, to a single cluster, suggesting that this clustering pattern is real and not an artifact of the analyses. Interestingly, under this scenario the identified clusters did not correspond to geographic location, with each cluster containing individuals sampled from all sites.

STRUCTURE and STRUCTURE HARVESTER analyses indicated that, for the northern long-eared bat, *K* = 3 had the highest probability (Fig 3). Similar to the little brown bat, the identified clusters did not correspond to geographic location. However, few individuals were assigned to each cluster with high probability, which may indicate cluster assignment is artificial rather than biologically meaningful.

**Fig 4:**
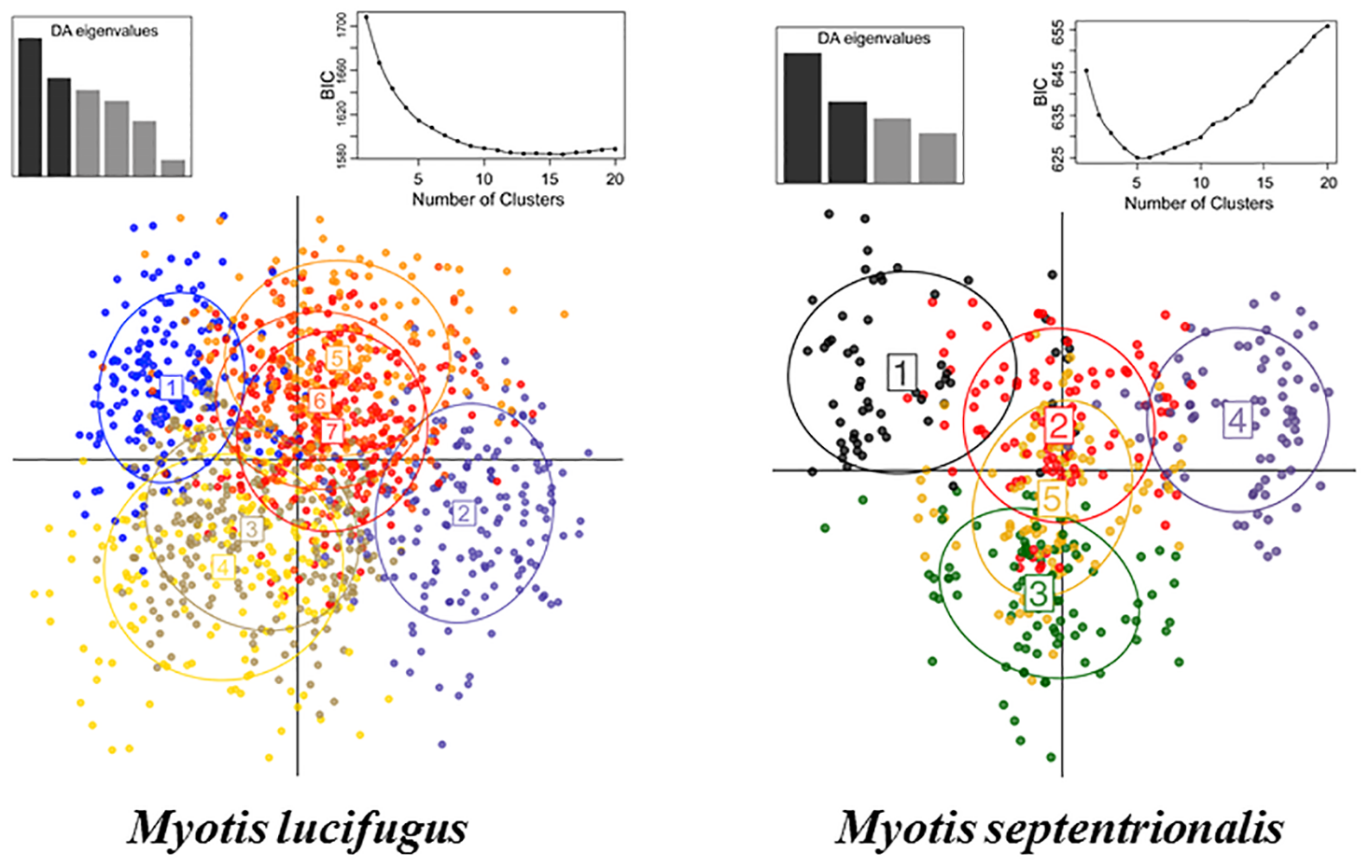
Little brown bat (*Myotis lucifugus*) and northern long-eared bat (*M*. *septentrionalis*) inference of population structure. Little brown bat and northern long-eared bat scatterplots of genetically distinct clusters identified through DAPC within adegenet. Also shown are the corresponding BIC plots for various numbers of clusters (above right) and the DA eigenvalues for the displayed scatterplots (below right).

For the little brown bat, initial examination of the loading plot during the DAPC in adegenet indicated that locus Mluc21 was heavily influencing the DAPC clustering, and therefore variable loading was not well distributed across loci. To address this, we removed Mluc21 from further analyses. Subsequently, the Bayesian Information Criterion (BIC) scores decreased until they plateaued at *K* = 12, although after *K* = 7 the change in BIC values was minimal (Fig 4). The *a*-score suggested the optimum number of principal components to retain was 26 with an *a*-score of 0.7. We plotted several values of *K* for analysis, and chose to present the plot for *K* = 7. The scatterplot shows that while clusters 1 and 2 appear to be somewhat separated from the remaining clusters, there is considerable overlap of all others. Examination of membership probabilities of individuals to particular clusters suggested that clusters were comprised of individuals across all sampled sites; therefore clusters were not concordant with geographic region or sampling location. While some individuals were asymmetrically assigned to single clusters with a relatively high probability (>0.80%), others had rather symmetric probability values that were divided among all clusters. DAPC conducted on 500 simulated data sets of unrelated individuals created for the little brown bat revealed one cluster, suggesting that clustering observed in our dataset is likely real and not an artifact.

The BIC scores revealed that *K* = 5 had the lowest BIC value for the northern long-eared bat (Fig 4). The *a*-score analysis suggested the optimal number of principal components to retain was 13 with an *a*-score of 0.7. The scatterplot of *K* = 5 revealed that three of five clusters show substantial overlap (Fig 4). This genetic similarity may explain the three clusters inferred using the STRUCTURE analysis (e.g., the DAPC clusters (1), (4), and (2, 3, 5) representing the STRUCTURE clusters 1, 2, and 3). Cluster assignment did not relate to geographic regions or sampling sites. As found in the little brown bat, the DAPC conducted on 500 simulated data sets for the northern long eared bat identified one cluster suggesting, the clustering observed in our dataset is not an artifact.

## Discussion

### Maternity Roost Site Fidelity

For summer maternity roosts, we found greater genetic differentiation of mtDNA than nDNA in both species. This likely suggests that genetic differentiation is driven by maternally-directed fidelity. Alternatively, microsatellites undergo high mutation rates; and the genetic differentiation identified in nDNA may be an artifact of size homoplasy [73]. However, similar patterns have been previously recorded for the little brown bat [9] and the northern long-eared bat [29]. Philopatry to summering sites may allow individuals to gain knowledge of resources including food and suitable roosts, which could increase survival [74,75]. For example, female Bechstein’s bats (*M*. *bechsteinii*) select sites with different microclimates through pregnancy and lactation [12]. Philopatry may also increase opportunities to form long-term bonds between individuals which may facilitate pup rearing as group living decreases thermoregulatory costs of mother and pup [76].

Philopatry should result in higher-than-expected relatedness among individuals within a summering site, if each site is inhabited by only a few maternal groups. Although our results indicate that summering site relatedness was not higher than random expectations, relatedness at summering sites was higher than at swarming sites. Combined, these data suggest that multiple maternal groups of unrelated individuals may share a single summering site. Similar evidence of philopatric behaviour towards summering colonies coupled with low values of relatedness has been measured in other bats including brown long-eraed bat (*Plecotus auritus*)[77] and Bechstein’s bats [78]. Because regional migrants exhibit temporal and geographical isolation of mating and birthing sites/events, and because swarming/hibernacula mating events can involve individuals from several summering areas, it is generally expected that any existing patterns of philopatry in bats will be most evident from mtDNA characteristics (e.g. [23,24]).

Our study and other recent genetic studies (e.g. [9,38]) suggest there is moderate mtDNA structuring among maternity colonies. However, capture-mark-recapture studies on the little brown bat suggest that this species exhibits strong year-to-year fidelity to these sites (e.g. [37]), which should lead to high mtDNA structuring. We suggest some explanations, which may lead to conflicting interpretations of behavioural and genetic signatures of philopatry. First, the origin of mtDNA haplotypes can pre-date the separation and/or origin of maternity colonies, resulting in the same haplotypes spread across multiple colonies. Secondly, even a small number of dispersing individuals can dilute the genetic signature of philopatry across sites. In addition, animals may be philopatric to an area but switch roosts through the summer e.g. [38], a characteristic that would also conceal mtDNA evidence of philopatry by mixing haplotypes among sites. These factors should be taken into consideration when interpreting genetic results, such as the genetic structuring found among sites, when complementary behavioural data is unavailable.

### Swarming Site Fidelity

Previous authors have suggested that weak nDNA structuring among summering colonies is a result of mating occurring away from summering colonies, where individuals from various summering sites congregate, facilitating gene flow [9, 78,79]. This congregation would result in high genetic diversity at swarming sites. We found greater genetic diversity at swarming sites than summering sites for the little brown bat and the northern long-eared bat. Analysis of nDNA and mtDNA data revealed that both species had only weak structure among swarming sites. These results are consistent with previous studies, such as Burns et al. [40], which found weak genetic structuring across little brown bat swarming sites. Weak nDNA structuring among swarming sites has been observed in other bat species including the Natterer’s bat (*M*. *nattereri*) [23] and brown long-eared bat [25]. Although both markers suggest weak structuring, there were higher levels of mtDNA structuring. This may be a result of individuals expressing some degree of site fidelity towards swarming sites; however the degree of fidelity may vary between individuals. This is consistent with the study of Norquay et al. [38] who identified both individual fidelity to swarming sites and inter-individual variation in little brown bats.

### Isolation by Distance

While no patterns of IBD were identified in the northern long-eared bat for summering or swarming sites, we found patterns of IBD in the little brown bat. Mitochondrial DNA haplotypes revealed IBD for swarming sites, as did nDNA across summering sites. The little brown bat swarming sites had a maximum distance of 869 km apart, while the northern long-eared bat swarming sites were 309 km apart. The lack of isolation by distance observed in the northern long-eared bat may therefore be an artifact of sampling sites being too close together relative to the potential movement range of the species to detect patterns of IBD. In the Appalachian Mountains, Miller-Butterworth et al. [41] reported similar patterns of female fidelity and isolation by distance towards hibernacula in the little brown bat, although it was suggested that movement restrictions were also a result of topography. An alternate explanation for female IBD may be explained by the maternal guidance hypothesis where females may show their offspring the routes or locations of sites [37, 80,81]. If young-of-the-year are restricted by distance and follow adult females during fall migration; females may reduce travel distances to meet the needs of young bats.

### Seasonal site connectivity

Within both species, haplotype diversity was significantly greater in swarming sites than summering sites, supporting the hypothesis that swarming sites are comprised of individuals from various summering sites. When we assessed genetic differentiation between swarming and summering sites, we identified high nDNA genetic similarity between the seasonal sites in both species. This weak structuring suggests high levels of gene flow across eastern mainland Canada. If swarming sites are the primary mating sites for both species, they are important for the maintenance of gene flow among summer colonies. Further examination of seasonal site connectivity using mtDNA data indicated complex structuring in both species. Analyses of the little brown bat mtDNA suggested that Quebec swarming sites show the greatest genetic differentiation from summering sites. Specifically, when compared to summering sites, two of the three Quebec sites (site 13: Mont Saint Hilaire, QC and site 14: Mine-aux-Pipistrelles, QC) were part of 11 of the 14 significant pairwise *F*_*ST*_ values. Analysis of northern long-eared bat mtDNA only suggested significant differentiation between seasonal sites relative to summering site 29 (Kejimkujik National Park, NS) and three swarming sites (site 6: Lear Shaft, NS; site 8: Rawdon Mine, NS; site 11: Howes Cave, NB). In comparison to the other summering sites sampled, site 29 is located in south-west of Nova Scotia, and is the most distant of the northern long-eared bat swarming sites sampled.

### Overall structure of nDNA

As predicted, a greater degree of genetic structuring was found in the northern long-eared bat than the little brown bat. Clusters identified for the little brown bat using DAPC had high degrees of genetic similarity and these results complemented those from STRUCTURE and collectively suggest low genetic structuring. In comparison, the five clusters identified for the northern long-eared bat through DAPC appeared to have greater genetic differentiation; however, there was a high degree of similarity between three of the five clusters. Genetic similarity between the three clusters may explain the three genetically differentiated clusters revealed by STRUCTURE. However, in both species, STRUCTURE and DAPC analyses revealed that membership probabilities were not correlated with the geographic location of sites. The factors driving the identified genetic structuring remain unclear and should be examined further.

We suggest there are three potential explanations for the observed patterns of population structure in these species. First, structuring observed for both species may be a remnant of historical factors. As identified by Burns et al. [40], little brown bats inhabiting eastern Canada appear to have experienced a population expansion following the last glaciation event, which occurred ≈12,000 years ago [82]. It is therefore possible that the little brown bat clusters are remnants of structure due to isolation of historical refugia. There is also a high likelihood that northern long-eared bats followed a similar expansion pattern to little brown bats in this region. As northern long-eared bats form smaller summering colonies and are highly forest dependent, a greater length of time may be required for the northern long-eared bat to have their genetic material redistributed. This may explain the greater amount of structuring observed in the northern long-eared bat. Alternatively, the observed patterns may result from complex behavioural/mating choices made by individuals that have yet to be clarified. For example, studies on black-backed woodpeckers (*Picoides arcticus*) identified fine-scale genetic structuring but a lack of structuring over a large spatial scale [83,84]. Within these studies isolation by distance was not detected, and it has been suggested that fine-scale structuring is likely a result of behaviour of individuals when dispersing to exploit new habitats [83]. Lecomte et al. [85] identified fine-scale genetic structuring coupled with evidence of high gene flow across large spatial scales in greater snow geese (*Chen caerulescens atlantica*). Here it was suggested that structuring resulted from varying degrees of female philopatry between rearing and nesting seasons. As our study sampled females across the summering season, we were unable to identify if females exhibit varying degrees of philopatry between stages of pregnancy and rearing offspring. Additionally, fine-scale structuring may be a result of sex-biased dispersal. If this hypothesis is tested and sexes are examined separately, one sex should exhibit fine-scale structuring while the other lacks structuring [86].

### Conclusion and conservation implications

Despite the behavioural differences between the little brown bat and northern long-eared bat, our study found great similarity in the population genetic structuring across seasonal sites for both species. In both species, the genetic differentiation between summering sites is likely explained by philopatry. Swarming sites appear to be composed of individuals from various summering sites, and in both species weak structuring is likely driven by varying degrees of fidelity among individuals. As well, we determined that the overall population structuring, observed in both species, is not explained by geographic location or distance between sites.

Migratory species, including bats, are at a heightened risk of decline due to their broad-scale movements and multiple habitat requirements. Management and conservation efforts require an adequate understanding of migratory processes, particularly in light of increased anthropogenic development. If any of the seasonal sites are disrupted or migratory barriers disconnect seasonal sites, local populations may experience local extinction. Within North America, many bat populations are declining as a result of White-nose Syndrome (WNS) resulting from the fungus *Pseudogymnoascus destructans*, which inhabits bat hibernacula [87]. Bat populations have been devastated, with millions of bat mortalities occurring across seven infected species, including the little brown bat and northern long-eared bat [87,88]. As different species often occupy the same hibernacula, understanding interspecific movement patterns may aid in predicting the spread and epidemiology of emergent infectious diseases such as WNS. Further research examining how population genetic structure varies among species affected by *P*. *destructans* is crucial to understanding the spread of this disease. As well, fatalities of bats have been globally documented at wind turbine facilities [89]. As the majority of wind turbine fatalities appear to occur during the fall migration, examination of migration patterns may further allow us to determine how to prevent bat mortality from wind turbines [90]. Analyses of genetic connectivity among seasonal sites can provide inferences on migratory connectivity and population structuring that may be overlooked using direct observations. Future work across these species ranges would provide insight into the connectivity among regional populations that is essential for the management of these wide ranging, regionally-migrating species.

## Supporting Information

S1 Table Numbers of individuals profiled per site. Number of mtDNA and nDNA samples analyzed for the little brown bat (*M*. *lucifugus*) and northern long-eared bat (*M*. *septentrionalis*) from each sample site.
(XLSX)

S2 Table Microsatellite PCR multiplex amplification information. Primer concentrations were the same for both the little brown bat (*M*. *lucifugus*) and northern long-eared bat (*M*. *septentrionalis*) unless indicated. See Burns et al. [59] for more information.
(XLSX)

S3 Table Mitochondrial haplotype frequencies obtained from the little brown bat (*Myotis lucifugus*) and northern long-eared bat (*M*. *septentrionalis*). (XLSX)

S4 Table Mitochondrial haplotype data characteristics for samples site and site type. This table includes the number of sequences obtained (*N*), number of haplotypes (*H*), haplotype diversity (*h*) with standard deviation (S.D.) and nucleotide diversity (*π*) with standard deviation (S.D.).
(XLSX)

S5 Table Pairwise mtDNA *F*_*ST*_ values between summering sites (a) and swarming sites (b) for the little brown bat (*Myotis lucifugus*) and northern long-eared bat (*M*. *septentrionalis*. Site numbers correspond with Fig 1. Pairwise *F*_*ST*_ values are located above diagonal and relative *p-values* are below the diagonal. Bolded values indicate statistically significant values after Bonferroni correction.
(XLSX)

S6 Table Pairwise mtDNA (a) and nDNA (b) *F*_*ST*_ values between summering and swarming sites for the little brown bat (*Myotis lucifugus*) and northern long-eared bat (*M*. *septentrionalis*. Site numbers correspond with Fig 1. Bolded values indicate statistically significant values after Bonferroni correction.
(XLSX)
