# Correction: Population Genetic Structure Within and among Seasonal Site Types in the Little Brown Bat (*Myotis lucifugus*) and the Northern Long-Eared Bat (*M*. *septentrionalis*)

**DOI:** 10.1371/journal.pone.0133457

**Published:** 2015-07-20

**Authors:** The PLOS ONE Staff

There are multiple errors in the fifth sentence of the Abstract. The publisher apologizes for the errors. The correct sentence is: Both species exhibited moderate degrees of mitochondrial DNA differentiation (little brown bat: *F*_*ST(SUMMER)*_ = 0.093, *F*_*ST(SWARMING)*_ = 0.052; northern long-eared bat: *F*_*ST(SUMMER)*_ = 0.117, *F*_*ST(SWARMING)*_ = 0.043) and little microsatellite DNA differentiation among summering and among swarming sites.
